# Increased Aquaporin-7 Expression Is Associated with Changes in Rat Brown Adipose Tissue Whitening in Obesity: Impact of Cold Exposure and Bariatric Surgery

**DOI:** 10.3390/ijms24043412

**Published:** 2023-02-08

**Authors:** Gema Frühbeck, Leire Méndez-Giménez, Sara Becerril, Beatriz Ramírez, Ana Wenting Hernández-Pardos, Javier A. Cienfuegos, Víctor Valentí, Rafael Moncada, Victoria Catalán, Javier Gómez-Ambrosi, Inês V. da Silva, Graça Soveral, Amaia Rodríguez

**Affiliations:** 1 Metabolic Research Laboratory, Clínica Universidad de Navarra, 31008 Pamplona, Spain; 2 Centro de Investigación Biomédica en Red Fisiopatología de la Obesidad y Nutrición (CIBEROBN), Instituto de Salud Carlos III, 28029 Madrid, Spain; 3 Obesity and Adipobiology Group, Instituto de Investigación Sanitaria de Navarra (IdiSNA), 31008 Pamplona, Spain; 4 Department of Endocrinology & Nutrition, Clínica Universidad de Navarra, 31008 Pamplona, Spain; 5 Department of Surgery, Clínica Universidad de Navarra, 31008 Pamplona, Spain; 6 Department of Anesthesia, Clínica Universidad de Navarra, 31008 Pamplona, Spain; 7 Research Institute for Medicines (iMed.ULisboa), Faculty of Pharmacy, Universidade de Lisboa, 1649-004 Lisbon, Portugal

**Keywords:** aquaglyceroporin, lipogenesis, peroxisome proliferator-activated receptor γ, adipocyte hypertrophy, sleeve gastrectomy

## Abstract

Glycerol is a key metabolite for lipid accumulation in insulin-sensitive tissues. We examined the role of aquaporin-7 (AQP7), the main glycerol channel in adipocytes, in the improvement of brown adipose tissue (BAT) whitening, a process whereby brown adipocytes differentiate into white-like unilocular cells, after cold exposure or bariatric surgery in male Wistar rats with diet-induced obesity (DIO) (*n* = 229). DIO promoted BAT whitening, evidenced by increased BAT hypertrophy, steatosis and upregulation of the lipogenic factors *Pparg2*, *Mogat2* and *Dgat1*. AQP7 was detected in BAT capillary endothelial cells and brown adipocytes, and its expression was upregulated by DIO. Interestingly, AQP7 gene and protein expressions were downregulated after cold exposure (4 °C) for 1 week or one month after sleeve gastrectomy in parallel to the improvement of BAT whitening. Moreover, *Aqp7* mRNA expression was positively associated with transcripts of the lipogenic factors *Pparg2*, *Mogat2* and *Dgat1* and regulated by lipogenic (ghrelin) and lipolytic (isoproterenol and leptin) signals. Together, the upregulation of AQP7 in DIO might contribute to glycerol influx used for triacylglycerol synthesis in brown adipocytes, and hence, BAT whitening. This process is reversible by cold exposure and bariatric surgery, thereby suggesting the potential of targeting BAT AQP7 as an anti-obesity therapy.

## 1. Introduction

Adipose tissue is classified into two main depots with different morphology, function and location: white (WAT) and brown (BAT) adipose tissue [1]. WAT stores energy in the form of triacylglycerol (TG) and secretes adipokines, while BAT oxidizes lipids to fuel adaptive thermogenesis [2]. Adipose tissue dysfunction is a hallmark of obesity [3]. During its pathological expansion in obesity, WAT undergoes profound morphological and functional changes, such as adipocyte hypertrophy, adipose tissue inflammation and fibrosis, as well as adipokine dysregulation [4]. A reduced BAT activity is also observed in the obese state, mainly as a result of the differentiation of brown adipocytes to white-like unilocular cells [5]. This process, known as “BAT whitening”, leads to a progressive brown adipocyte dysfunction, evidenced by increased inflammation, cell death, reduced vascularization, as well as impaired glucose uptake and non-shivering thermogenesis [5,6,7,8].

**Figure 1:**
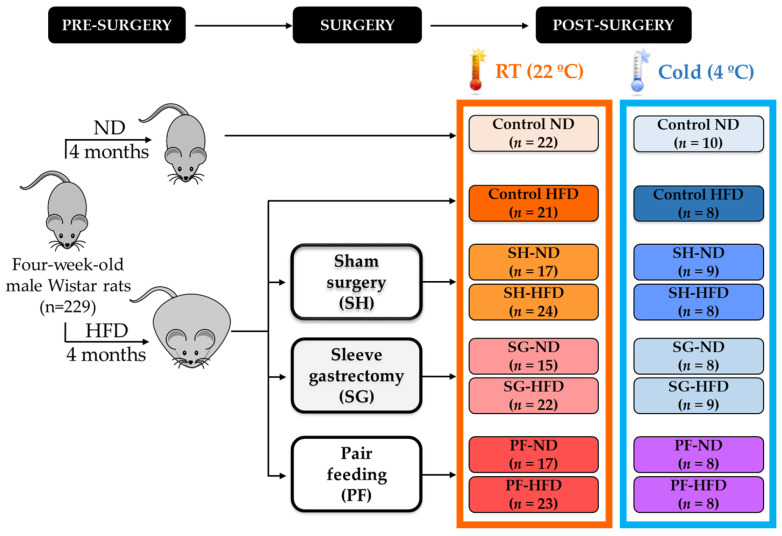
Experimental design. The experimental groups are extensively explained in Section 4.1.

Mature adipocytes are the result of the terminal differentiation of adipose progenitors and committed preadipocytes, in a process called adipogenesis [9]. The transcription factor PPARγ is the master regulator for determining the phenotype and function of brown and white adipocytes during adipogenesis [10]. Aquaporin-7 (AQP7) is a glycerol channel with key functions in adipocyte differentiation and growth [11]. *Aqp7* gene promoter presents PPAR response elements (PPRE) [12] and is markedly increased during white adipocyte differentiation [13,14]. Under basal conditions, AQP7 is locked to the lipid droplet by perilipin-1 in order to prevent its translocation to the plasma membrane and subsequent glycerol efflux [15]. In response to lipolytic factors, such as catecholamines or leptin, AQP7 reduces the complex formation with perilipin, translocates to the plasma membrane and facilitates glycerol transport [13,16,17,18]. Accordingly, the defective glycerol release in *Aqp7*-KO mice results in intracellular glycerol accumulation, which is used for de novo TG synthesis, leading to adipocyte hypertrophy [19,20]. Thus, the altered expression of AQP7 in WAT is associated with obesity and metabolic disorders, such as type 2 diabetes [16,21,22,23,24,25,26]. Literature about the function of AQP7 in BAT is scarce, but supports its role in brown adipogenesis. Cortisone, a glucocorticoid promoting preadipocyte conversion to mature adipocytes [27], as well as dietary n-3 long-chain polyunsaturated fatty acids (PUFA) eicosapentaenoic acid (EPA) and docosahexaenoic (DHA) [28] upregulate AQP7 in brown adipocytes from rodents. However, the impact of obesity on AQP7 expression in BAT and its metabolic consequences remain unknown. We hypothesized that AQP7 participates in the improvement of BAT whitening of rats with diet-induced obesity (DIO) 1 week after cold exposure and/or 1 month after bariatric surgery (Figure 1).

## 2. Results

### 2.1. Cold Exposure Improved Metabolic Profile and BAT Whitening in DIO Rats

Cold exposure for 1 week reduced (all *p* < 0.05) the increased markers of adiposity (body weight, whole-body adiposity and the adipokine leptin) (Table 1). Moreover, markers of insulin resistance (hyperinsulinemia and HOMA index) and lipid profile (free fatty acids [FFA], triacylglycerols and total cholesterol) were also improved in rats with DIO exposed to cold (Table 1). An increase (*p* < 0.05) in orexigenic hormone ghrelin was also observed during cold acclimation (Table 1).

**Figure 2:**
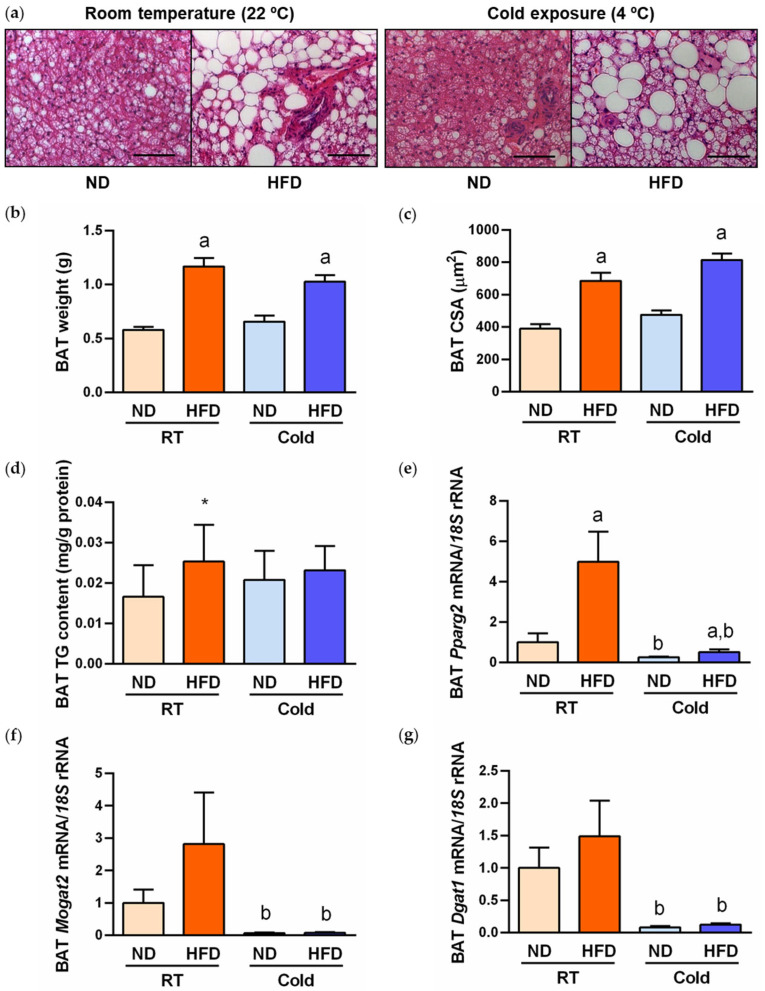
Protective effect of cold exposure against BAT whitening by reducing triacylglycerol content. (**a**) Representative hematoxylin–eosin-stained images of BAT whitening in rats fed either ND or HFD at RT and 1 week after cold exposure (magnification 200×, scale bar = 100 µm). BAT weight (**b**), cell surface area (CSA) (**c**), triacylglycerol content (**d**) and mRNA expression of the lipogenic transcription factor *Pparg2* (**e**) and the effector lipogenic enzymes *Mogat2* (**f**) and *Dgat1* (**g**). The transcript levels in rats fed ND at RT was assumed to be 1. Statistical differences were evaluated by two-way ANOVA or one-way ANOVA followed by Tukey’s post hoc test in case of interaction between factors. ^a^ *p* < 0.05 effect of diet; ^b^ *p* < 0.05 effect of cold exposure; * *p* < 0.05 vs. rats fed ND.

HFD feeding increased (*p* < 0.05) BAT weight (Figure 2b) and brown-to-white conversion, evidenced by increased brown adipocyte cell size (Figure 2a,c) and intracellular TG content (Figure 2d). Previous studies have shown that pharmacological PPARγ activation significantly accelerates TG synthesis and promotes hypertrophy in brown adipocytes [29]. This process is associated with upregulated absorption of FFA and elevated synthesis of TG using either monoacylglycerol (MAG) or glycerol-3-phosphate as the initial acyl acceptors [30]. The monoacylglycerol *O*-acyltransferase-2 (MOGAT2) is the enzyme that catalyzes the first step in the TG synthesis, where MAG is converted to diacylglycerol (DAG). Then, TGs are directly synthesized from DAG by the diacylglycerol *O*-acyltransferase 1 (DGAT1) enzyme. Transgenic and pharmacological studies in mice have demonstrated the beneficial effects of MOGAT2 inhibition as a therapy for several metabolic diseases, including obesity and insulin resistance [30,31]. Interestingly, the transcription factor PPARγ directly regulates MOGAT2 and DGAT1 promoter activity. Thus, due to their metabolic relevance, we next studied the gene expression of these lipogenic factors, finding an upregulated expression of the lipogenic transcription factor *Pparg2* and its downstream molecules *Mogat2* and *Dgat1* in rats fed the HFD (Figure 2e–g). Cold acclimation for 1 week did not change BAT weight or adipocyte area (Figure 2a–c), but prevented the increase in TG content in parallel with the downregulation (*p* < 0.05) of *Pparg2*, *Mogat2* and *Dgat1* transcript levels (Figure 2d–g).

### 2.2. AQP7 Expression Is Increased during BAT Whitening and Downregulated by Cold Exposure

**Figure 3:**
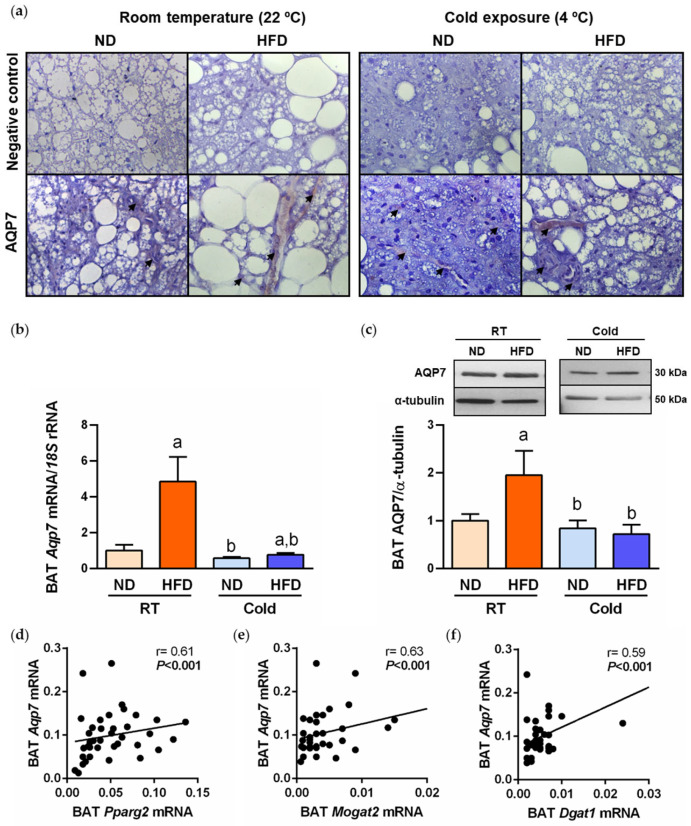
The expression of AQP7 in BAT is increased during BAT whitening and downregulated by cold exposure. (**a**) Representative images of AQP7 immunostaining in sections of BAT from rats fed either ND or HFD at RT and 1 week after cold exposure (magnification 400×); negative controls without primary antibody are shown in the *upper panels* and arrows indicate specific staining of AQP7 in the *bottom panels*. Gene (**b**) and protein (**c**) expression levels of AQP7 in BAT of experimental animals. The transcript and protein levels in rats fed ND at RT were assumed to be 1. Correlation of *Aqp7* mRNA and lipogenic factors *Pparg2* (**d**), *Mogat2* (**e**) and *Dgat1* (**f**) mRNA levels. Statistical differences were evaluated by two-way ANOVA. ^a^ *p* < 0.05 effect of diet; ^b^ *p* < 0.05 effect of cold exposure.

We first explored the histological distribution of AQP7 in BAT by immunohistochemistry. As illustrated in Figure 3a, the immunolabeling of AQP7 was mainly detected in endothelial cells lining the vascular wall, and a weaker labeling was also observed in mature brown adipocytes. Next, we investigated the impact of DIO and cold acclimation in AQP7 expression in BAT by real-time PCR and Western blot. An upregulation of AQP7 mRNA and protein expression (both *p* < 0.05) was observed in parallel to BAT whitening induced by DIO (Figure 3b,c). Accordingly, univariate correlation analysis showed a positive association between *Aqp7* mRNA and transcript levels of lipogenic factor *Pparg2*, *Mogat2* and *Dgat1* (Figure 3d–f), as well as with plasma glycerol (r = 0.20, *p* = 0.006). In the multiple lineal regression analysis, whole-body adiposity contributed independently to 27% (*p* < 0.001) of AQP7 protein levels in BAT after controlling for body weight, suggesting the strong impact of obesity in the expression of AQP7 in this thermogenic fat depot. By contrast, AQP7 mRNA and protein were markedly repressed during cold acclimation (Figure 3b,c).

### 2.3. Sleeve Gastrectomy Ameliorates BAT Whitening under Both Thermic Conditions

As expected, sleeve gastrectomy significantly (all *p <* 0.05) reduced body weight, total white adiposity and the adipokine leptin, as well as markers of insulin resistance (insulin and HOMA), independently of the diet provided after surgical intervention to the experimental animals (Table 2). Since this bariatric surgery procedure removes the gastric fundus, the main production site of ghrelin [32], a dramatic decrease (*p <* 0.0001) in post-surgical levels of this orexigenic hormone was observed. Moreover, a reduction (both *p* < 0.05) in serum glycerol and triacylglycerols was detected after sleeve gastrectomy. Cold exposure further ameliorated insulin sensitivity and lipid profile of the surgical groups (Table 2). By contrast, cold acclimation increased circulating FFA and leptin of experimental animals submitted to sleeve gastrectomy compared to the sham surgical group, probably as a consequence of β-adrenergic stimulation in adipocyte lipolysis [33].

**Figure 4:**
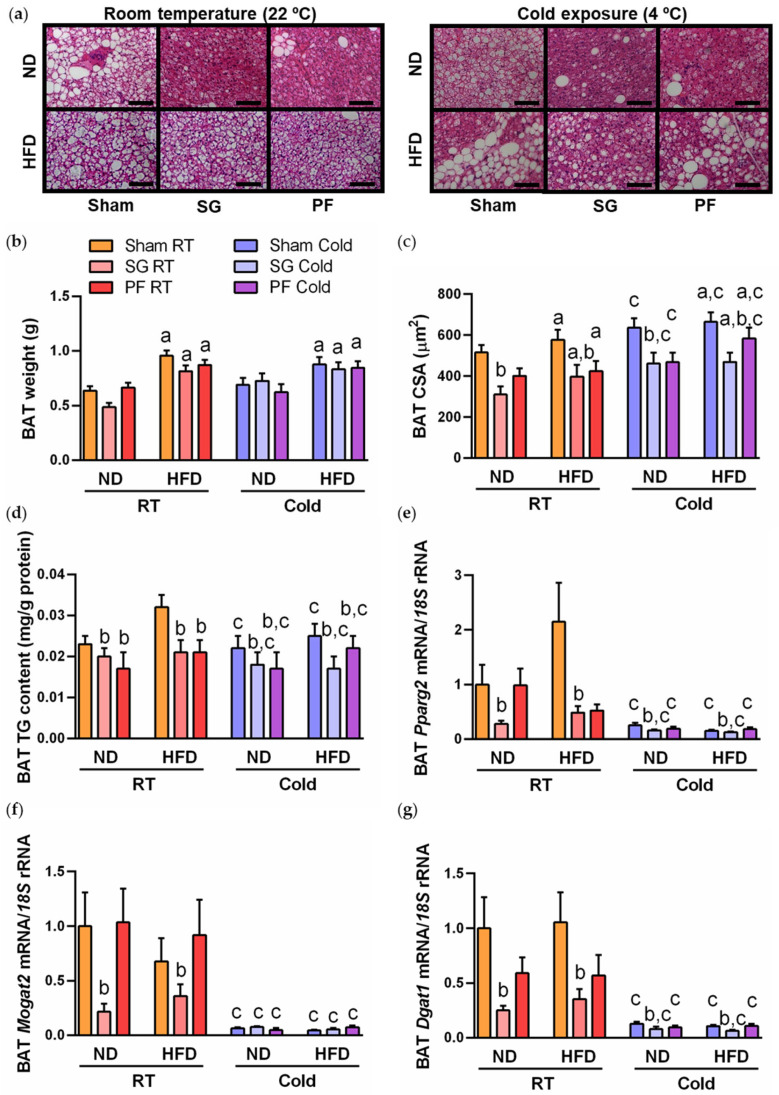
Beneficial effect of sleeve gastrectomy on BAT whitening in DIO rats by reducing adipocyte hypertrophy and triacylglycerol content under different thermic conditions. (**a**) Representative images of BAT whitening after surgical or dietary interventions in animals exposed to different thermic conditions (magnification 200×, scale bar = 100 µm). BAT weight (**b**), cell surface area (CSA) (**c**), triacylglycerol content (**d**) and mRNA expression of lipogenic transcription factor *Pparg2* (**e**) and effector lipogenic enzymes *Mogat2* (**f**) and *Dgat1* (**g**). Transcript levels in rats submitted to sham surgery fed ND at RT was assumed to be 1. Statistical differences were evaluated by three-way ANOVA. ^a^ *p* < 0.05 effect of diet; ^b^ *p* < 0.05 effect of sleeve gastrectomy; ^c^ *p* < 0.05 effect of cold exposure.

Sleeve gastrectomy did not change BAT weight (Figure 4b), but improved BAT morphology and function, as shown by the reduction in brown adipocyte hypertrophy (Figure 4a,c) and steatosis (Figure 4d), together with the repression of the transcription of the lipogenic genes *Pparg2*, *Mogat2* and *Dgat1* (Figure 4e–g). Similar results were observed in rats acclimated to cold, with the exception of gene expression of *Mogat2*, which remained unchanged after sleeve gastrectomy (Figure 4f). The increase in mean BAT CSA in response to cold exposure in DIO rats (Figure 4a,c) was due to the augment in the frequency of adipocytes of medium (CSA between 200 and 900 µm^2^, *p* < 0.05) or large (CSA = 900–1000 µm^2^ or higher, *p* < 0.01) size, without changes in adipocytes of small size (CSA ≤ 100 µm^2^, *p* = 0.613).

### 2.4. A Reduction in AQP7 Expression Is Found after Weight Loss Achieved by Sleeve Gastrectomy

**Figure 5:**
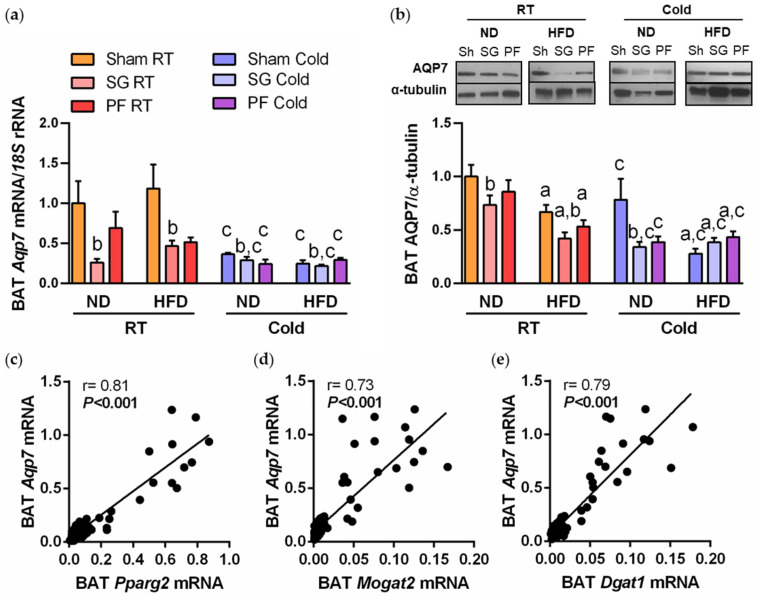
AQP7 expression in BAT was repressed after sleeve gastrectomy in DIO rats under both thermic conditions. Gene (**a**) and protein (**b**) expression levels of AQP7 in BAT after surgical, dietary and thermic interventions. Correlation of *Aqp7* mRNA and lipogenic factors *Pparg2* (**c**), *Mogat2* (**d**) and *Dgat1* (**e**) mRNA levels. Statistical differences were evaluated by two-way ANOVA. ^a^ *p* < 0.05 effect of diet; ^b^ *p* < 0.05 effect of cold exposure; ^c^ *p* < 0.05 effect of cold exposure.

To further confirm the potential association of AQP7 expression with changes in BAT whitening observed after sleeve gastrectomy, we assessed the transcript and protein levels of this glycerol channel in BAT of the experimental groups under both thermic conditions. Significant downregulations of mRNA and protein levels of AQP7 (both *p* < 0.05) were observed in BAT of DIO rats submitted to sleeve gastrectomy (Figure 5a,b), even in rats exposed to cold, which exhibited lower initial AQP7 transcript and protein levels (both *p* < 0.05). These changes were not observed in the pair-fed groups, suggesting that the impact of bariatric surgery in AQP7 expression in BAT is beyond caloric restriction. As observed in rats with DIO, post-surgical changes in *Aqp7* mRNA were strongly associated with transcripts of *Pparg2*, *Mogat2* and *Dgat1* (Figure 5c–e). The switch of DIO rats from HFD to ND also downregulated (*p* < 0.05) AQP7 protein expression probably as a result of the reduction in BAT whitening in these experimental animals (Figure 5b).

### 2.5. Regulation of AQP7 in Brown Adipocytes Is Regulated by Lipolytic and Lipogenic Factors

**Figure 6:**
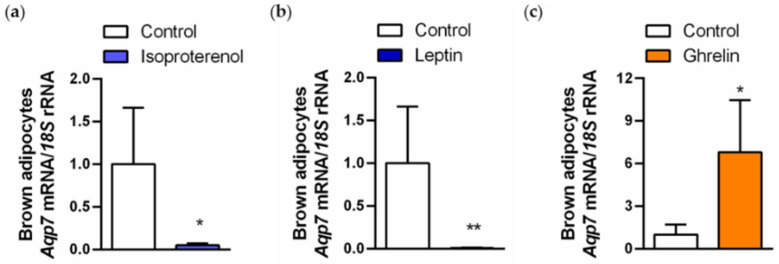
Regulation of *Aqp7* gene expression in rat brown adipocytes. Effect of lipolytic (**a**,**b**) and lipogenic (**c**) factors on the mRNA expression of *Aqp7* in brown adipocytes. Statistical analysis was assessed by Student’s *t* test. * *p* < 0.05; ** *p* < 0.01 vs. unstimulated cells.

Since our results point to a strong association of AQP7 with the regulation of lipid metabolism in BAT, we assessed the effect of lipolytic (isoproterenol and leptin) and lipogenic (ghrelin) factors on the regulation of AQP7 in rat brown adipocytes. The stimulation of differentiated rat brown adipocytes with isoproterenol (10 µmol/L) or leptin (10 ng/mL) dramatically downregulated (both *p* < 0.05) *Aqp7* expression (Figure 6a,b), while ghrelin (100 pmol/L) upregulated (*p* < 0.05) the transcript levels of this aquaglyceroporin (Figure 6c).

## 3. Discussion

**Figure 7:**
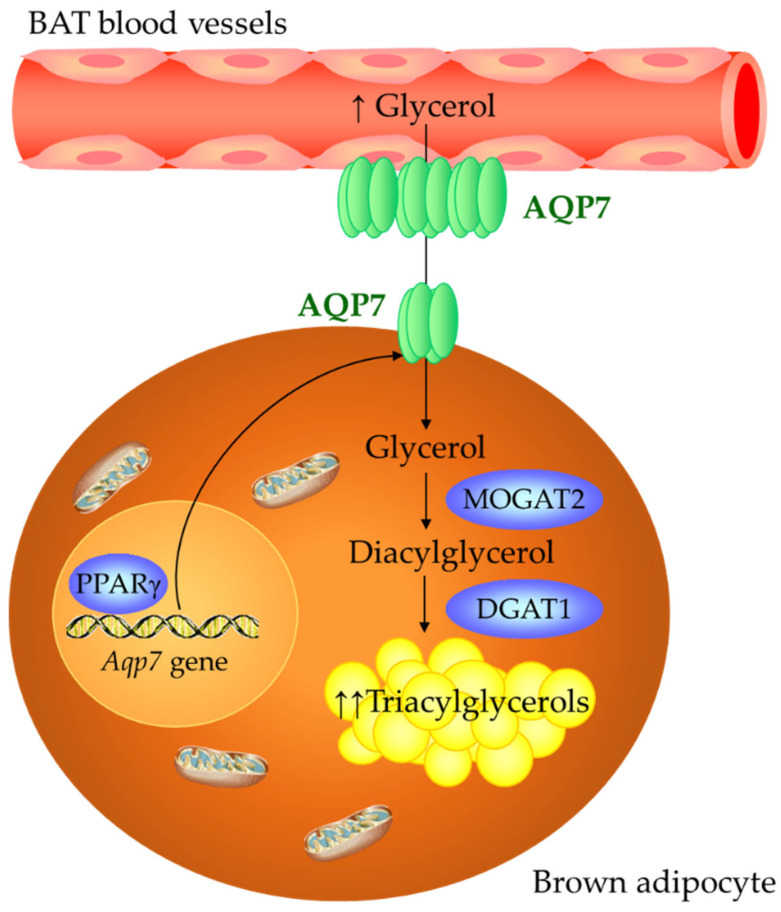
Role of AQP7 in BAT whitening.

Obesity is a multifactorial disease resulting from a chronic imbalance between energy intake and energy expenditure, which are simultaneously under the broader influence of environmental factors as well as genetics and epigenetics [34]. In this regard, a conceptual model linking global warming and the obesity epidemic is emerging [35], where the global rise in temperature directly influences obesity via a concomitant reduction in BAT activity [34,36]. In genetic or acquired obesity, the decline in BAT function involves the regulation of several transcription factors leading to the loss of thermogenic phenotype and the gain of a WAT-like phenotype [5,37], but the molecular mechanisms underlying this process remain poorly understood. In the present study, we revealed the association of AQP7 with changes in BAT whitening (Figure 7) in DIO rats challenged with environmental, dietary and surgical stimuli. Our experimental model of DIO developed BAT whitening, evidenced by brown adipocyte hypertrophy, steatosis and increased expression of lipogenic enzymes. AQP7 allows the bidirectional movement of water and glycerol across plasma membranes [38,39], with glycerol permeation being a central element of fat accumulation and the pathophysiology of obesity [16,19,20]. It has been proposed that, in the adipose tissue, glycerol permeability through AQP7 is regulated via trafficking (e.g., catecholamine/insulin-dependent subcellular re-organization of AQP7 in adipocytes) [16,40] or pH [39,41]. Using immunohistochemistry, we herein confirmed previous studies [14,42,43,44,45,46,47], showing that AQP7 expression is mainly found in capillary endothelial cells lining the vascular wall. Skowronski and colleagues found that both insulin resistance and fasting increase the expression of AQP7 in capillary endothelia in the adipose tissue [42], supporting its role in facilitating glycerol transport under different metabolic conditions. Although to a lesser extent, AQP7 was also detected in rat mature brown adipocytes. Diet-induced obesity was associated with an increase in circulating glycerol levels as well as with an upregulation of AQP7 in BAT, which was strongly associated with the increase in the lipogenic factors *Pparg2*, *Mogat2* and *Dgat1*. Low expression of AQP7 has been also found in murine beige adipocytes, which is downregulated throughout beige adipocyte differentiation [46]. Thus, we proposed that the AQP7 increase during BAT whitening facilitates glycerol transport between capillary endothelial cells and brown adipocytes, where glycerol is used as a substrate for de novo lipogenesis (Figure 7). Although AQP7 is considered the main glycerol channel in the adipose tissue, aquaglyceroporins AQP3, 9 and 10 [16,40,48], orthodox AQP5 [49] and superaquaporin AQP11 [50,51] also represent additional pathways for the transport of glycerol in adipocytes. Further investigations analyzing the role of other AQPs expressed in BAT are warranted.

BAT activation has been proposed as an attractive therapeutic target for the prevention of obesity and its metabolic alterations. Acute cold exposure increases β-adrenergic activation in BAT, activating the lipolysis of TG stored in brown adipocytes and liberating FFA, the major fuel substrates for thermogenesis in experimental animals and humans [52,53]. In addition, BAT takes up large amounts of circulating glucose and lipids that are used for thermogenesis. Accordingly, we observed a reduction in adiposity together with an improvement of insulin resistance and the altered lipid profile in DIO rats in response to 7 days of cold challenge (4 °C) compared to normal housing conditions (22 °C). Short-term cold exposure is associated with pronounced changes in pathways involved in the remodeling of TG and glycerophospholipids in BAT [54]. In our study, a reduction in BAT steatosis without changes in brown adipocyte cell size was observed after cold challenge, which is in accordance with previous reports [5]. In line with this observation, we found a dramatic repression in the transcription of lipogenic factors *Pparg2*, *Mogat2* and *Dgat1* in BAT after short-term exposure to cold. Interestingly, these changes induced by cold were accompanied by a significant downregulation of the glycerol channel AQP7 in BAT. Moreover, a strong positive association was detected between transcript levels of *Aqp7* and those of the lipogenic genes *Pparg2*, *Mogat2* and *Dgat1* in BAT from cold-acclimated rats. Taken together, the downregulation of AQP7 might reflect the extensive changes in the expression of genes involved in glycerolipid metabolism during cold adaptation in BAT.

The low metabolic activity of BAT found in obesity can be also reversed after successful weight reduction by bariatric surgery [55,56,57,58,59]. Our group [55,56] and others [60] found that sleeve gastrectomy, a restrictive bariatric surgical procedure, improves BAT morphology and function through the upregulation of genes related to brown adipocyte differentiation and thermogenesis (*Prdm16*, *Dio2* and *Ucp1*). As expected, we observed a reduction in whole-body adiposity and insulin resistance after sleeve gastrectomy in DIO rats. This metabolic amelioration was accompanied by a reduction in BAT whitening, revealed by a decrease in brown adipocyte area, steatosis and transcription of the lipogenic factors *Pparg2*, *Mogat2* and *Dgat1.* By contrast, the switch to ND in the sham-operated animals only improved BAT weight and CSA, without significant changes in the expression of lipogenic factors, supporting that the beneficial effects of sleeve gastrectomy on BAT whitening are beyond caloric restriction. Interestingly, we observed a marked repression of AQP7 after sleeve gastrectomy in DIO rats exposed to dietary and environmental temperature challenges, which was also robustly associated with changes in the transcription of factors related to lipogenesis. Thus, changes in BAT expression of AQP7 after bariatric surgery might reflect the improved brown adipocyte function by reversing WAT-like phenotype found in obesity.

Modulation of AQP7 expression by hormones is well documented in the literature [61]. In white adipocytes, several stimuli involved in the regulation of lipolysis, such as catecholamines, leptin, atrial natriuretic peptide, uroguanylin and guanylin [12,16,17,62,63], and lipogenesis, including insulin, ghrelin, dexamethasone or follicle-stimulating hormone (FSH) [64,65,66,67], regulate AQP7 expression. We herein show that in brown adipocytes, AQP7 is negatively regulated by isoproterenol and leptin, which are involved in the β-adrenergic activation of BAT and adipocyte lipolysis. The negative regulation of AQP7 by both lipolytic factors suggests a negative feedback regulation to restrict glycerol release from BAT during states of sympathoactivation, such as cold exposure. By contrast, we found that ghrelin positively regulated AQP7 expression in brown adipocytes, which appears to facilitate glycerol influx to be used as a substrate for the maintenance of TG stores in these thermogenic cells. 

In conclusion, our data provide new insight into the presence and function of AQP7 in brown adipocytes. AQP7 is associated with changes in BAT whitening, based on its strong correlation with factors involved in lipogenesis as well as its regulation by lipogenic (ghrelin) and lipolytic (isoproterenol and leptin) signals. The upregulation of AQP7 in diet-induced obesity might contribute to glycerol influx used for TG synthesis, and hence, brown adipocyte hypertrophy (Figure 7). By contrast, cold exposure and bariatric surgery, which are well-known activators of BAT function, downregulate AQP7 in parallel to the improvement of BAT whitening. Further investigations are required to establish the suitability of the regulation of AQP7 in BAT as a therapeutic target for human obesity-associated type 2 diabetes. 

## 4. Materials and Methods

### 4.1. Experimental Animals and Study Design

Four-week-old male Wistar rats (*n* = 229) obtained from the breeding house of the University of Navarra were housed in individual cages and maintained under pathogen-free conditions, controlled temperature (22 ± 2 °C), relative humidity (50 ± 10%) and on a 12:12 light–dark cycle (lights on at 08:00 am). Animals were fed ad libitum for 4 months with either a normal diet (ND) (*n* = 32) (12.1 kJ: 4% fat, 82% carbohydrate and 14% protein, diet 2014S, Harlan, Teklad Global Diets, Harlan Laboratories Inc., Barcelona, Spain) or a high-fat diet (HFD) (*n* = 197) (23.0 kJ/g: 59% fat, 27% carbohydrate and 14% protein, diet F3282; Bio-Serv, Frenchtown, NJ, USA) (Figure 1). Body weight and food intake were registered weekly. DIO rats were randomly assigned into 3 weight-matched groups: (i) sleeve gastrectomy (resecting 60% of total gastric volume) (*n* = 54); (ii) sham surgery without gastric resection (*n* = 58); and (iii) pair-feeding that received the same amount of food eaten by the gastrectomized group (*n* = 56). Anesthesia, sham surgery and sleeve gastrectomy were performed according to previously described methodology [23]. Following the surgical interventions, DIO rats were fed either ND or HFD. Moreover, 3 weeks after surgery, a group of rats were exposed to cold (4 °C, *n* = 68) for 1 week in order to evaluate BAT function, whereas the rest of animals remained at RT (22 °C, *n* = 161) [5,54]. Four weeks after the surgical and dietary interventions, rats were killed by decapitation after an 8-h fasting period. All experimental procedures conformed to the European Guidelines for the care and use of Laboratory Animals (directive 2010/63/EU) and were approved by the Ethical Committee for Animal Experimentation of the University of Navarra (049/10).

### 4.2. Blood Analysis

Blood samples were immediately collected and centrifuged at 700× *g* at 4 °C for 15 min to obtain sera samples. Serum glucose was determined by an automatic glucose sensor (Ascencia Elite, Bayer, Barcelona, Spain). Total ghrelin (#EZRGRT-91 K, Millipore, Billerica, MA, USA), leptin and insulin (#90030 and #90060, Crystal Chem, Inc., Chicago, IL, USA) were determined by ELISA [68]. Intra- and inter-assay coefficients of variation were 0.8% and 2.8% for total ghrelin, 5.4% and 6.9% for leptin, and 3.5% and 6.3% for insulin. Insulin resistance was calculated using the homeostasis model assessment (HOMA), calculated with the following formula: fasting insulin (μU/mL) × fasting glucose (mmol/L)/22.5). Serum glycerol (F6428, Sigma, St. Louis, MO, USA), FFA (434-91795 and 436-91995, WAKO Chemicals, GmbH, Neuss, Germany) and TG (TR22421, Infinity^TM^, Thermo Scientific, Melbourne, Australia) were measured by enzymatic methods using commercially available kits.

### 4.3. Sample Handling

BAT and WAT were carefully dissected out, weighed, snap-frozen and stored at −80 °C until RNA and protein extraction. Total white adiposity was calculated as the sum of epididymal, perirenal and subcutaneous fat pad weights. A small portion of BAT was fixed in 4% formaldehyde for histological analyses. Another portion of BAT was used for the isolation of adipocytes and stromal vascular fraction cells (SVFCs) by collagenase digestion. Total RNA isolation and purification from BAT and brown adipocytes were performed using the QIAzol^®^ Reagent (Qiagen, Hilden, Germany) and the RNeasy Lipid Tissue Mini Kit (Qiagen), according to the manufacturer’s instructions. Total proteins from BAT were extracted using RIPA buffer (0.1% SDS, 1% Triton X-100, 5 mmol/L EDTA·2H_2_O, 1 mol/L Tris, 150 mmol/L NaCl, 1% sodium deoxycholate, pH 7.40) supplemented with a protease inhibitor cocktail (Complete^TM^ Mini-EDTA free, Roche, Mannheim, Germany). Samples were centrifuged at 16,000× *g* at 4 °C for 15 min and total protein concentration was quantified by the Bradford assay (Bio-Rad Laboratories, Inc., Hercules, CA, USA) using bovine serum albumin (BSA) (Sigma) as standard. 

### 4.4. Brown Adipocyte Culture and Treatment

Interscapular BAT (500 mg) was minced and incubated for 30 min at 37 °C with constant shaking in adipocyte medium (DMEM/F-12 [1:1] (11320-033, Invitrogen, Paisley, U.K.), 17.5 mmol/L glucose, 16 µmol/L biotin, 18 µmol/L panthotenate, 100 µmol/L ascorbate and antibiotic-antimycotic (A5955, Sigma) supplemented with 2% BSA (Sigma) and 1 mg/mL collagenase A (Roche). The digestion was stopped adding adipocyte medium supplemented with 10% newborn calf serum (NCS) (Sigma) and suspended cells were then filtered through a 100 µm nylon cell strainer (BD Biosciences, Erembodegem, Belgium) and centrifuged at 600× *g* for 10 min at RT [69]. Adipocytes were decanted and SVFCs were resuspended in adipocyte medium supplemented with 10% NCS. SVFCs from BAT were seeded at 2 × 10^5^ cell/cm^2^ and grown in adipocyte medium supplemented with 10% NCS. After 4 days, the medium was changed to adipocyte medium supplemented with 10% NCS, 20 nmol/L insulin (Sigma) and 2 nmol/L triiodothyronine (T3) (Sigma) in order to induce brown adipocyte differentiation [70]. Cells were fed every 2 days with the same medium for the remaining 10 days of brown adipocyte differentiation. Differentiated rat brown adipocytes were serum-starved for 24 h and then treated with isoproterenol (10 µmol/L) (Sigma), leptin (10 nmol/L) (PeproTech EC, Inc., Rocky Hill, NJ, USA) or acylated ghrelin (100 pmol/L) (Tocris, Ellisville, MO, USA) for 24 h. One sample per experiment was used to obtain control responses in the presence of the solvent.

### 4.5. Real-Time PCR

Transcript levels for aquaporin 7 (*Aqp7*), monoacylglycerol *O*-acyltransferase 2 (*Mogat2*), diacylglycerol *O*-acyltransferase 1 (*Dgat1*) and peroxisome proliferator-activator receptor γ 2 (*Pparg2*) were quantified by real-time PCR (7300 Real-Time PCR System; Applied Biosystems, Foster City, CA, USA). Primers and probes (Table S1) were designed using the software Primer Express 2.0 (Applied Biosystems) and were synthesized by Sigma. Primers or TaqMan^®^ probes encompassing fragments of the areas from the extremes of two exons were designed to ensure the detection of the corresponding transcript avoiding genomic DNA amplification. BLAST analysis (https://blast.ncbi.nlm.nih.gov/Blast.cgi) was applied to evaluate the specificity of the sequences of primers and probes. The thermal cycling conditions included an initial denaturation at 95 °C for 10 min followed by 45 cycles at 95 °C for 15 s and extension at 59 °C for 1 min, using the TaqMan^®^ Universal PCR Master Mix (Applied Biosystems). The primer and probe concentrations were 300 and 200 nmol/L, respectively. All reactions were performed in duplicate and expression levels were normalized to *18S* rRNA (Applied Biosystems) for each sample, and relative quantification was calculated using the 2^−∆∆Ct^ formula [21]. 

### 4.6. Western Blot

BAT protein samples (30 μg) were denatured and separated by SDS-PAGE in Any kD™ Mini-PROTEAN^®^ TGX^TM^ Precast Gels (Bio-Rad) at 200 V for up to 45 min and transferred to polyvinylidene difluoride (PVDF) membranes (Bio-Rad) using the Trans-Blot Turbo transfer system (Bio Rad) at 2.5 A for 10 min. Blots were blocked in Tris-buffered saline (10 mmol/L Tris-HCl, 150 mmol/L NaCl, pH 8.00) with 0.05% Tween 20 (TBS-T) containing 5% non-fat dry milk for 1 h at RT. Blots were then incubated overnight at 4 °C with rabbit polyclonal anti-AQP7 (sc-28625, Santa Cruz Biotechnology, Inc., Santa Cruz, CA, USA) or murine monoclonal anti-α-tubulin (T6793, Sigma) antibodies (diluted 1:1000 and 1:5000, respectively, in blocking solution). The antigen–antibody complexes were visualized using horseradish peroxidase-conjugated anti-rabbit or anti-mouse IgG antibodies (diluted 1:5000 in blocking solution) and the Pierce^TM^ ECL Plus Western-blotting Substrate (Thermo Scientific) using the ChemiDoc MP imaging system (Bio-Rad). Band density was quantified using the Image Lab 6.0 software (Bio-Rad) and values were normalized to α-tubulin from the same lanes. Representative images for AQP7 and α-tubulin are shown; all the bands for each picture come always from the same gel, although they may be spliced for clarity. Uncropped blots have been provided as Supporting Information files at the time of initial submission.

### 4.7. Immunohistochemistry of AQP7

The indirect immunoperoxidase method was used to detect AQP7 in histological sections of BAT [16]. Sections (4 µm) of formalin-fixed, paraffin-embedded BAT were dewaxed in xylene, rehydrated in decreasing concentrations of ethanol and treated with 3% H_2_O_2_ (Sigma) in absolute methanol for 10 min at RT to quench endogenous peroxidase activity. Slides were blocked for 30 min with 1% murine serum (Sigma) diluted in Tris-buffered saline (TBS) (50 mmol/L Tris, 0.5 mol/L NaCl; pH 7.36) to prevent nonspecific adsorption. Sections were incubated overnight at 4 °C with rabbit polyclonal anti-AQP7 (sc-28625, Santa Cruz) antibody diluted 1:100 in TBS. After three washes in TBS (5 min each), slides were incubated with DAKO Real^TM^ EnVision^TM^ anti-rabbit/mouse (K5007; Dako, Golstrup, Denmark) for 1 h at RT. The peroxidase reaction was visualized using a 0.5 mg/mL diaminobenzidine (DAB)/0.03% H_2_O_2_ solution diluted in 50 mmol/L Tris-HCl, pH 7.36, and Harris hematoxylin solution (Sigma) as counterstaining. Negative control slides without primary antibodies were included to assess non-specific staining.

### 4.8. Measurement of BAT Intracellular Triacylglycerols

Intracellular TG content was measured by enzymatic methods using the Infinity^TM^ Triglycerides Liquid Stable Reagent (Thermo Scientific), as previously described [71]. Briefly, BAT samples were homogenized and diluted in saline at a final concentration of 50 mg/mL. BAT homogenates were diluted (1:1) in 1% deoxycholate (Sigma) and incubated at 37 °C for 5 min. Afterwards, samples were diluted 1:100 in the Infinity™ Triglycerides Liquid Stable Reagent (Thermo Scientific) and incubated for 30 min at 37 °C. The resulting dye was measured based on its absorbance at 550 nm. Concentrations were determined compared with a standard curve of triglycerides (Infinity™ Triglycerides Standard, Thermo Scientific). The protein content of the preparations was measured by the Bradford assay. All assays were performed in duplicate.

### 4.9. Determination of the Area of Brown Adipocytes

The cell surface area (CSA) of brown adipocytes was measured as previously described [23]. Briefly, biopsies of BAT were fixed in 4% formaldehyde, embedded in paraffin, cut into sections of 4 μm and stained with hematoxylin–eosin. Images of three fields per section from each animal were captured with the 20× objective, and the brown adipocyte CSA from, at least 100 cells/section was measured using the software AxioVision Release 4.6.3 (Zeiss, Göttingen, Germany).

### 4.10. Statistical Analysis

Statistical analyses were performed using the SPSS 15.0 software. Data are expressed as the mean ± SEM. The normality of the variables of the study was assessed by the Kolmogorov–Smirnov and Shapiro–Wilk tests. Statistical differences between mean values were analyzed using three-way, two-way or one-way ANOVA followed by Tukey’s post hoc test, where appropriate. Pearson correlation coefficients (*r*) and stepwise multiple linear regression analysis were used to analyze the association between variables. A *p* value < 0.05 was considered statistically significant.

## Figures and Tables

**Table 1:** Body composition and metabolic profile in DIO rats after cold exposure for 1 week.

	RT (22 °C)		Cold (4 °C)	
	ND	HFD	ND	HFD
Body weight (g)	481 ± 13	619 ± 14 ^a^	474± 20 ^b^	551± 22 ^a,b^
Total white adiposity (g)	16.5 ± 2.1	38.7 ± 2.2 ***	17.8 ± 2.3	28.9 ± 2.7 *^,†^
Glucose (mg/dL)	80 ± 2	90 ± 2 ^a^	81 ± 3	87 ± 4 ^a^
Insulin (µU/mL)	1.8 ± 0.4	3.7 ± 0.4 ^a^	1.6 ± 0.6 ^b^	1.9 ± 0.7 ^a,b,^
HOMA	0.37 ± 0.10	0.84 ± 0.10 ^a^	0.32 ± 0.15 ^b^	0.42 ± 0.17 ^a,b^
FFA (mmol/L)	25.4 ± 1.7	21.0 ± 1.8	17.9 ± 2.4 ^b^	17.5 ± 2.5 ^b^
Glycerol (mg/dL)	28.8 ± 2.4	33.3 ± 2.5 ^a^	22.5 ± 3.4	34.4 ± 3.8 ^a^
Triacylglycerol (mg/dL)	119 ± 12	100 ± 12	40 ± 17 ^b^	44 ± 19 ^b^
Total cholesterol (mg/dL)	105 ± 7	115 ± 7	66 ± 10 ^b^	95 ± 11 ^b^
Total ghrelin (ng/mL)	0.9 ± 0.1	0.7 ± 0.1	1.1 ± 0.1 ^b^	1.3 ± 0.2 ^b^
Leptin (ng/mL)	7.7 ± 1.1	17.1 ± 1.2 ^a^	6.4 ± 1.6 ^b^	10.1 ± 1.8 ^a,b^

RT, room temperature; ND, normal diet; HFD, high-fat diet; HOMA, homeostasis model assessment; FFA, free fatty acids. Differences between groups were analyzed by two-way ANOVA or one-way ANOVA followed by Tukey’s test in case of interaction between factors. ^a^ *p* < 0.05 effect of diet; ^b^ *p* < 0.05 effect of cold exposure; * *p* < 0.05; *** *p* < 0.001 vs. rats fed ND; † *p* < 0.05 vs. same group at RT.

**Table 2:** Effect of diet, sleeve gastrectomy and cold exposure on body composition and metabolic profile of rats with DIO.

RT (22 °C)						
	ND			HFD		
	Sham	SG	PF	Sham	SG	PF
Body weight (g)	503 ± 10	478 ± 11 ^b^	495± 11	549± 12 ^a^	514 ± 13 ^a,b^	554± 12 ^a^
Total white adiposity (g)	21.8 ± 1.5	17.5 ± 1.6 ^b^	24.2 ± 1.4	28.8 ± 2.0 ^a^	27.2 ± 2.3 ^a,b^	30.0 ± 2.3 ^a^
Glucose (mg/dL)	79 ± 3	78 ± 3	90 ± 3	101 ± 3 *	103 ± 4 *	111 ± 3 *
Insulin (µU/mL)	2.2 ± 0.2	1.8 ± 0.2 ^b^	1.4 ± 0.3	2.2 ± 0.3	1.8 ± 0.3 ^b^	2.3 ± 0.3
HOMA	0.43 ± 0.05	0.21 ± 0.06 ^b^	0.43 ± 0.06	0.55 ± 0.6 ^a^	0.46 ± 0.7 ^a,b^	0.73 ± 0.06 ^a^
FFA (mmol/L)	19.6 ± 1.2	18.8 ± 1.3 ^b^	19.8 ± 1.2	22.3 ± 1.5 ^a^	19.7 ± 1.5 ^a,b^	21.3 ± 1.5 ^a^
Glycerol (mg/dL)	23.9 ± 1.6	21.4 ± 1.7 ^b^	24.2 ± 1.7	28.9 ± 2.0 ^a^	25.1 ± 2.0 ^a,b^	29.9 ± 2.0
Triacylglycerol (mg/dL)	101 ± 5	61 ± 5 ^b^	76 ± 5	69 ± 6 ^a^	53 ± 6 ^a,b^	68 ± 6 ^a^
Total cholesterol (mg/dL)	110 ± 3	101 ± 8	109 ± 3	93 ± 4 ^a^	82 ± 4 ^a^	88 ± 4 ^a^
Total ghrelin (ng/mL)	1.0 ± 0.7	0.5 ± 0.1 ^b^	1.1 ± 0.1	0.9 ± 0.1 ^a^	0.4 ± 0.1 ^a,b^	0.9 ± 0.1 ^a^
Leptin (ng/mL)	7.4 ± 0.7	5.0 ± 0.8 ^b^	9.0 ± 0.8	12.4 ± 0.9 ^a^	9.9 ± 1.0 ^a,b^	16.0 ± 0.9 ^a^
**Cold (4 °C)**						
	**ND**			**HFD**		
	**Sham**	**SG**	**PF**	**Sham**	**SG**	**PF**
Body weight (g)	503 ± 10	477 ± 19 ^b^	514± 13 ^a,b^	558± 18 ^a^	527± 17 ^a,b^	557± 21 ^a^
Total white adiposity (g)	20.5 ± 1.7	17.0 ± 2.3 ^b^	18.3 ± 1.8	29.2 ± 1.8 ^a^	25.1 ± 1.7 ^a,b^	27.8 ± 2.3 ^a^
Glucose (mg/dL)	88 ± 5	76 ± 5	77 ± 5	87 ± 5	75 ± 5 ^#^	81 ± 5
Insulin (µU/mL)	1.5 ± 0.3 ^c^	1.3 ± 0.4 ^c^	0.9 ± 0.4 ^c^	1.4 ± 0.4 ^c^	1.4 ± 0.4 ^c^	1.7 ± 0.4 ^c^
HOMA	0.33 ± 0.08 ^c^	0.23 ± 0.10 ^b^	0.16 ± 0.09 ^b^	0.31 ± 0.09 ^c^	0.25 ± 0.09 ^a,b,c^	0.34 ± 0.09 ^a,c^
FFA (mmol/L)	16.9 ± 2.0 ^c^	18.3 ± 2.1 ^c^	17.9 ± 2.1 ^c^	15.5 ± 2.1 ^c^	16.5 ± 2.0 ^c^	19.4 ± 2.1 ^c^
Glycerol (mg/dL)	18.2 ± 2.7	22.1 ± 2.8	22.4 ± 2.8	27.1 ± 2.8 ^a^	25.9 ± 2.7 ^a^	31.2 ± 2.8 ^a^
Triacylglycerol (mg/dL)	44 ± 8 ^c^	53 ± 8 ^c^	37 ± 8 ^c^	53 ± 6 ^c^	41 ± 7 ^c^	44 ± 8 ^c^
Total cholesterol (mg/dL)	68 ± 5 ^c^	81 ± 5 ^c^	68 ± 5 ^c^	88 ± 5 ^c^	74 ± 5 ^c^	72 ± 5 ^c^
Total ghrelin (ng/mL)	1.3 ± 1.1 ^c^	1.0 ± 0.1 ^b,c^	1.5 ± 0.1 ^c^	1.2 ± 0.1 ^a,c^	0.9 ± 0.1 ^a,b,c^	1.3 ± 0.1 ^c^
Leptin (ng/mL)	5.6 ± 1.3 ^c^	6.1± 1.3 ^c^	4.0 ± 1.4 ^c^	8.8 ± 1.3 ^a,c^	8.8 ± 1.3 ^a,c^	9.0 ± 1.3 ^a,c^

RT, room temperature; ND, normal diet; HFD, high-fat diet; HOMA, homeostasis model assessment; FFA, free fatty acids. Differences between groups were analyzed by three-way ANOVA or one-way ANOVA followed by Tukey’s test in case of interaction between factors. ^a^ *p* < 0.05 effect of diet; ^b^ *p* < 0.05 effect of bariatric surgery; ^c^ *p* < 0.05 effect of cold exposure; * *p* < 0.05 vs. rats fed ND; ^#^ *p* < 0.05 vs. sham surgery group.

## Data Availability

The data presented in this study are available upon reasonable request from the corresponding author. The data are not publicly available due to privacy restrictions.

## Supplementary Materials

The following supporting information can be downloaded at: https://www.mdpi.com/article/10.3390/ijms24043412/s1.
