# Supplementary material for: Increased Aquaporin-7 Expression Is Associated with Changes in Rat Brown Adipose Tissue Whitening in Obesity: Impact of Cold Exposure and Bariatric Surgery
Source: Int J Mol Sci. 2023 Feb 8;24(4):3412. doi: 10.3390/ijms24043412 (PMC9963055; doi:10.3390/ijms24043412)
Supplement: Supplementary file 1 [file ijms-24-03412-s001.zip › ijms-2104151-supplementary.pdf]

Table S1. Sequences of primers and TaqMan® probes.

Gene (GenBank accesion no.)	Oligonucleotide sequence (5′-3′)	Nucleotides
Aqp7		
(NM_019157.2)		
Forward	GGCTTCGTGGATGAGGTATTTG	724-745
Reverse	ACAGTCCAGCACTTCAAGGGAC	794-815
Taqman® probe	FAM-AGCTGTGTATCTTCGCCATCACG-TAMRA	761-783
Dgat1		
(NM_053437.1)		
Forward	CGGTCCCCAACCATCTGATAT	1048-1068
Reverse	TTTCCACTCATGTCTCAATGCTGTGGCA	1132-1152
Taqman® probe	FAM-TTCCACTCATGTCTCAATGCTGTGGCA-TAMRA	1091-2018
Mogat2		
(NM_001109436.2)		
Forward	TCCCTGTCTCTTTGGTCAAGACA	289-311
Reverse	TTCTTAACCTGTGCACTGAAAGCA	382-405
Taqman® probe	FAM-CGGAACTACATCGCAGGCTTTCACCC-TAMRA	330-355
Pparg2		
(NM_013124)		
Forward	CTGACCCAATGGTTGCTGATTAC	257-279
Reverse	CCTGTTGTAGAGTTGGGTTTTTTCA	351-375
Taqman® probe	FAM-TGAAGCTCCAAGAATACCAAAGTGCG-TAMRA	290-315

Aqp7, aquaporin-7; *Dgat1,* diacylglycerol *O*-acyltransferase 1; *Mogat2,* monoacylglycerol *O*-acyltransferase 2; *Pparg,* peroxisome proliferator-activator receptor γ 2.
